# A Triple Threat? The Role of Diet, Nutrition, and the Microbiota in T1D Pathogenesis

**DOI:** 10.3389/fnut.2021.600756

**Published:** 2021-04-01

**Authors:** Emma E. Hamilton-Williams, Graciela L. Lorca, Jill M. Norris, Jessica L. Dunne

**Affiliations:** ^1^The University of Queensland Diamantina Institute, The University of Queensland, Woolloongabba, QLD, Australia; ^2^Microbiology and Cell Science Department, Genetics Institute, Institute of Food and Agricultural Science, University of Florida, Gainesville, FL, United States; ^3^Department of Epidemiology, Colorado School of Public Health, University of Colorado Anschutz Medical Campus, Aurora, CO, United States; ^4^Janssen Research and Development, Raritan, NJ, United States

**Keywords:** type 1 diabetes, microbiota, diet, probiotics, disease risk

## Abstract

In recent years the role of the intestinal microbiota in health and disease has come to the forefront of medical research. Alterations in the intestinal microbiota and several of its features have been linked to numerous diseases, including type 1 diabetes (T1D). To date, studies in animal models of T1D, as well as studies in human subjects, have linked several intestinal microbiota alterations with T1D pathogenesis. Features that are most often linked with T1D pathogenesis include decreased microbial diversity, the relative abundance of specific strains of individual microbes, and altered metabolite production. Alterations in these features as well as others have provided insight into T1D pathogenesis and shed light on the potential mechanism by which the microbiota plays a role in T1D pathogenesis, yet the underlying factors leading to these alterations remains unknown. One potential mechanism for alteration of the microbiota is through diet and nutrition. Previous studies have shown associations of diet with islet autoimmunity, but a direct contributing factor has yet to be identified. Diet, through introduction of antigens and alteration of the composition and function of the microbiota, may elicit the immune system to produce autoreactive responses that result in the destruction of the beta cells. Here, we review the evidence associating diet induced changes in the intestinal microbiota and their contribution to T1D pathogenesis. We further provide a roadmap for determining the effect of diet and other modifiable factors on the entire microbiota ecosystem, including its impact on both immune and beta cell function, as it relates to T1D. A greater understanding of the complex interactions between the intestinal microbiota and several interacting systems in the body (immune, intestinal integrity and function, metabolism, beta cell function, etc.) may provide scientifically rational approaches to prevent development of T1D and other childhood immune and allergic diseases and biomarkers to evaluate the efficacy of interventions.

## Introduction

T1D is an autoimmune disease hallmarked by the inability of the body to produce insulin due to the autoimmune destruction of the beta cells in the endocrine pancreas. While the specific underpinnings of the pathogenesis of T1D are largely unknown, there is a large heritability component, and more than 50 genetic loci have been associated with an increase in T1D risk (1). In recent years, the development of more specific genetic risk scores (GRS) have been able to refine T1D risk into a continuous variable. The most novel of these GRS, the GRS2, has shown great discriminatory power in future prediction of T1D (2). However, given the polygenic nature of T1D along with the idea that there are likely other factors or triggers contributing to T1D initiation and progression, these GRS are highly sensitive but not specific, meaning, a large proportion of individuals with a high GRS2 score will never go on to develop T1D.

A major advancement in the field was defining the staging paradigm for T1D, noting that, in individuals with a high risk of developing T1D, i.e., those with a high HLA genetic risk or a family history of T1D, development of two or more islet autoantibodies (stage 1) carried a lifetime risk of developing T1D close to 100% and further refinement of this population based on dysglycemia (stage 2) resulted in an even higher relative risk of T1D (3). However, given the large variability in progression to T1D even in these refined populations, the differentiation between why some individuals progress more rapidly than others is largely unknown and is thought to involve environmental factors. In fact, in recent years, the largest relative increases in T1D in the United States have been in African American and Hispanic youth, further suggesting an increasing importance for environmental factors contributing to T1D, as these populations are considered to carry lower genetic risk (4). Similarly, an earlier report also showed that the largest increase in T1D in Australia was among those with lower HLA risk, suggesting a larger influence of environmental factors (5).

Indeed, in 2004, The Environmental Determinants of Diabetes in the Young (TEDDY) was launched to specifically study the environmental factors contributing to T1D pathogenesis (6). While no single factor has been found, a multitude of factors have been shown to contribute to islet autoimmunity (IA) and/or T1D. Many of these factors have direct or indirect contributions to either the diet or the gut microbiota in children and will be discussed in this review. Additional studies, both in humans and mouse models of T1D, have further implicated the role of both diet and microbiota in T1D pathogenesis.

One potential mechanism that has been implicated in T1D pathogenesis is that of increased intestinal permeability, or a leaky gut. Evidence from both rodent models (7, 8) and humans (9, 10) suggest that intestinal permeability is increased in T1D, suggesting a potential pathogenic mechanism. However, the direct mechanisms by which gut leakiness may contribute to T1D pathogenesis are poorly understood, but may include inflammation, immune responses and a loss of tolerance to β-cell antigens driven by changes in the microbiota.

Herein, we summarize the current studies describing the interplay of genetics, diet, and the microbiota on T1D development and possibilities to alter any one or a combination of these factors to modify T1D progression.

## Environmental Factors That Induce Microbiota Changes and Their Potential Role in T1D Pathogenesis

Seminal studies in T1D animal models, such as Non-Obese Diabetic mice (NOD) and Bio Breeding diabetes prone rat (BB-DP), have shown distinct changes in microbiota following the onset of diabetes. In Bio Breeding rats a higher abundance of *Bacteroides* was found in BB-DP rats, while higher concentrations of *Lactobacillus* and *Bifidobacterium* were correlated with diabetes resistant rats (BB-DR). The apparent natural dysbiosis status observed in BB-DR and BB-DP rats correlated with earlier reports of antibiotic-induced dysbiosis. The treatment of BB-DP rats with antibiotics prevented T1D onset (11, 12) while the incidence of T1D in a germ-free environment or following antibiotic treatment accelerated autoimmunity in NOD mice (13–17). Altogether, these data indicate the critical role of the microbiota in the development/onset of T1D.

Due to the high genetic heterogeneity and variable dietary patterns in human populations, the identification of diabetogenic or tolerogenic microbiota in T1D has not been straightforward. An initial study performed in stool samples collected by the Diabetes and Prevention study (DIPP) in Finland identified several bacterial genera that were more abundant in children that have seroconverted when compared to controls (18). Four matched case-control children with high-risk HLA-DQ genotype were selected, and three time points were analyzed, the last time point being seroconversion (presence of at least two islet autoantibodies). It was found that as the cases progressed to seroconversion, a decline in Firmicutes and an increase in Bacteroidetes was observed. At the seroconversion time point, the abundance of five Bacteroidetes species were significantly more abundant in cases than in controls (18). This study also suggested that a higher abundance of bacteria producing the short chain fatty acid (SCFA) butyrate- were present in controls. A similar decrease in microbial diversity was found to be associated with seroconversion in a study performed in 18 subjects from the TRIGR and FINDIA cohorts (19). It was also found that the high abundance of lactate and butyrate producing bacteria was negatively correlated to the number of islet autoantibodies in these cohorts. Kostic et al. (20) evaluated the DIABIMMUNE cohort to identify a possible microbial signature prognostic of T1D onset and potential links of this signature to metabolites in serum and stool. This densely sampled longitudinal study performed in 33 HLA-matched Estonian and Finnish infants (from birth to 3 years of age) compared the alpha diversity of the gut microbiota across time in non-converter (not seroconverted), seroconverted (not diagnosed with T1D), and T1D cases (seroconverted subjects diagnosed with T1D) (20). A significant difference in diversity was found prior to diagnosis of T1D in seroconverters that progressed to clinical disease. The decrease in diversity observed in T1D cases was correlated with high abundance of the *Rikenellaceae* family, as well as *Blautia, Ruminococcus*, and *Streptococcus* genera (20). While these studies were all relatively small, their results provided the initial evidence of a shift in the microbiota composition preceding T1D diagnosis.

Recently, The Environmental Determinants of Diabetes in the Young (TEDDY) study reported their findings on the metagenomes of a much larger multicenter study, which included three clinical locations in Europe (Finland, Germany, Sweden) and three in the United States (Colorado, Washington, Georgia/Florida) (21, 22). The goal of this specific study was to identify fluctuations in the microbiota from children starting at 3 months of age, to elucidate perturbations that may precede IA and T1D diagnosis (21, 22). This study offered the advantage of analyzing metagenomes as well as 16S rRNA gene community profiling. The metagenome data allowed a detailed pathway analyses and a better assignment of specific species that may contribute to the variance observed. The study found that the majority of variability in the metagenomes was explained by inter-subject differences, followed by age, geographical location, and breastfeeding of the subjects. A strong association was found with the ingestion of human milk and the abundance of *Bifidobacterium* species during the early stages of gut colonization (21, 22). The association is probably driven by the presence of human milk oligosaccharides (HMOs) that are almost predominantly degraded by *Bifidobacterium*, therefore acting as prebiotics (23). Furthermore, while *Bifidobacterium* species have been used as probiotics for decades, their role in prevention of T1D onset has not been explored in either animal models of T1D or in clinical trials. The analyses of the TEDDY IA cases and controls indicated a greater presence of *Lactobacillus rhamnosus* and *Bifidobacterium dentium* in controls, while IA cases had a higher abundance of *Streptococcus* species (*mitis, oralis*, and *pneumoniae*) (21). Similarly, the analyses of T1D cases and controls indicated controls had higher abundancy of lactic acid producing bacteria (*Lactococcus lactis* and *Streptococcus thermophilus*) commonly found in cheese and yogurt, while the T1D cases had a higher abundancy of *Bifidobacterium pseudocatenulatum, Roseburia hominis*, and *Alistipes shahii* (21). Detailed metabolic pathway analyses between cases and controls indicated that pathways involved in the synthesis of SCFA (e.g., acetate, butyrate, and propionate) are enriched in control subjects (21). In a separate study of adults with long standing T1D, higher levels of *Christensenella* associated with T1D and correlated negatively with fecal acetate (24). Together, these findings suggest the protective effect of SCFA, as previously reported in rodent models as well as other human T1D cohorts (21, 24–26).

Altered intestinal permeability has also been implicated in T1D pathogenesis. In an early study of individuals at various stages of disease, intestinal permeability was shown to be increased compared to controls, suggesting that the small intestine was somehow implicated in T1D pathogenesis (27). More recent studies have correlated gut microbiota changes to the changes in intestinal permeability and gut barrier proteins, specifically showing an associated decrease in SCFA-producing bacteria (28, 29). While a direct cause-and-effect has not been shown, there is increasing evidence that augmentation with SCFA-producing bacteria in individuals at-risk of developing T1D holds promise to change the trajectory of disease.

## Genetic Factors Contribute to Shaping The Gut Microbiota Linked to T1D

The establishment of a dysbiotic microbiota such as that found in T1D may have multiple causes. External factors such as diet, which provides different substrates for the bacteria to survive can profoundly influence microbiota composition (30). There may be an altered exposure to different communities of bacteria particularly during early life establishment of the microbiota. Furthermore, an altered host environment due to genetic polymorphisms may influence the intestinal environment or immune response to the bacteria. Finally, there can be purely stochastic effects driving variability between individuals (31). In general, it is considered that environmental factors have a stronger influence over the microbiota structure than host genetics (32), with heritability typically having weak effect sizes and few genes reaching genome-wide significance (32–35). Of interest, genetic polymorphism within the lactase gene shows a genome-wide association with the abundance of the *Bifidobacterium* genus (36), which was recently validated in a meta-analysis of 18,340 individuals (33). Lactase is a brush-border enzyme responsible for digestion of lactose from human and dairy derived milk (37). This suggests a significant diet-genetic-microbiota interaction for colonization of this important health-associated genera which could be relevant to the altered abundance of bifidobacteria seen in some studies of T1D risk (21). When looking at genetic polymorphisms directly linked to T1D, several studies have observed a relationship between T1D susceptibility alleles and an altered gut microbiota. A Swedish study of 403 children found that T1D-risk HLA alleles were significantly associated with gut microbiome composition (*P* = 0.01), with a range of taxa linked to specific HLA-risk groups (38). In a NOD mouse study, disease protection mediated by the presence of a protective MHC-II allele was found to be dependent on the gut microbiota (39). Similarly, genetic polymorphisms in the interleukin-2 pathway that are strongly linked to T1D risk in both humans and NOD mice, were found to be associated with an altered gut microbiota (40). Notably protective *IL2* alleles were linked to increased regulatory T cell frequency and mucin production within the intestine and IL2 therapy could mimic this effect on the gut suggesting that immunotherapy outcomes in T1D may also be linked to the gut microbiota response (40, 41). Together, these studies indicate that host genetics may play a role in shaping the intestinal niche that supports a dysbiotic microbiota in T1D and this could be a barrier to therapies reliant on colonization of new species into gut in individuals with T1D. If host genetics are a barrier, then manipulation of the microbiota may be better achieved early in life, with repeated delivery or combined with immunotherapy, for therapeutic benefit.

## Diet Induced Microbiota Changes in T1D

The gut microbiota is established and maintained by the host's genetics and external environmental factors, which include diet. Previous studies have shown that dietary factors are associated with the development of IA or with progression of IA to T1D, but a direct contributing factor has yet to be identified (42). One potential pathway by which diet may increase risk of T1D is by impacting the microbiota. Diet, through introduction of antigens and alteration of the microbiota and microbial metabolites, may influence the immune system to produce or to control autoreactive responses that result in the destruction of the beta cells. Here, we review the dietary factors that have been implicated in IA and T1D and discuss the evidence regarding their ability to induce changes in the intestinal microbiota and how this may contribute to T1D pathogenesis. While several dietary factors have been implicated in T1D, we will focus on those that have been found to be consistently (and with strong study designs) associated with IA and T1D, or progression from IA to T1D.

### Breast-Feeding and Infant Diet

Ever since the publication of the ecologic study linking insufficient breast-feeding to T1D incidence over time (43), numerous studies have been conducted on breast-feeding and T1D, and still the role of breastfeeding initiation, and whether an optimal duration of breastfeeding exists, remains unclear. Prospective studies of children at increased risk for T1D have reported inconsistent results regarding the association between breast-feeding and T1D (44–47). A large, population-based study of two birth cohorts in Norway suggests that while initiation of breastfeeding may reduce the risk of T1D, among those who were breastfed there does not appear to be a benefit of prolonging breastfeeding on the risk of T1D (48).

While the findings regarding breastfeeding and T1D may be equivocal, breast milk has a profound impact on the gut microbiota of the infant, as reviewed in Li et al. (49). Breastfeeding could lead to a healthier infant gut microbiota at an early stage of life compared with infants who are never breastfed (50, 51). As mentioned previously, the TEDDY study (21, 22) found that breast milk was the most significant factor associated with the structure of the developing microbiota. Current breast-feeding was associated with higher levels of *Bifidobacterium species* (*B. breve, B. bifidum*, and *B. longum*). *B. longum* subsp. *infantis* is a particularly important degrader of human milk oligosaccharide (HMO), and a genetic strain that is specific for the utilization of HMO within a subset of *B. longum* was present only in samples of breast-fed infants in TEDDY (21). Similarly, in a small Korean study, an increase in *Bifidobacterium* was observed in newborn infants that were breast-fed, whereas an increase in *Klebsiella* and *Serratia* was detected in newborn infants fed infant formula, suggesting a beneficial impact of breast milk (52).

The cessation of breast-feeding results in a functional shift in the gut microbiota (49) and a faster maturation of the gut (22). However, even with this shift, the impact of breastfeeding on the microbiota is still seen in 6-year old children, suggesting a long-term impact of exposure to breastmilk. The relative abundance of genera belonging to *Firmicutes* and *Actinobacteria* (and in particular *Bifidobacterium*, the predominant genus of *Actinobacteria*), was significantly different at 6 years of age between children that had been exclusively breast-fed for 4 months and those that had not (53). *Bifidobacterium* are important species in the healthy gut microbiota that can partially breakdown complex starches and oligosaccharides, making them available via “cross-feeding” to support butyrate producing bacterial populations such as *Roseburia* sp., *Eubacterium hallii*, and *Anaerostipes caccae* (54).

Age at introduction of complementary foods in infants has been examined regarding IA and T1D risk. Prospective observational studies suggest that late introduction of cereals (46) or gluten (47) is associated with increased risk of T1D and IA, respectively. However, an intervention in which gluten introduction was delayed to 12 months, vs. 6 months, showed no effect on IA or T1D risk (55, 56). It has been hypothesized that introduction of complementary foods impacts health by altering the microbiota. Introduction of any complementary foods at 3 months of age (vs. later) was associated with increased *Bilophila wadsworthia* and *Roseburia* in the infant's gut microbiota after adjustment for breast-feeding (57). However, others suggest that that the maturation of the gut microbiota is driven by the cessation of breast milk rather than the introduction of complementary foods (22). Using samples collected from 44 children from the German BABYDIET study, Endesfelder et al. showed that children with the most complex diet at 6-months of age were dominated by *Bacteroides* and this *Bacteroides* dominated community was associated with later development of IA. This suggested that fast-tracking of a more complex and adult-like microbial community may be related to establishment of taxa associated with T1D risk (58).

### Early Childhood Intake of Gluten and Fiber

Researchers have long been interested in gluten intake as a risk factor for T1D due to the similarities between and co-occurrence of T1D and celiac disease in individuals and families. The Diabetes Autoimmunity Study in the Young (DAISY) examined early childhood intake of gluten and did not find an association with either development of IA or progression from IA to T1D (59). Related to gluten intake is the intake of dietary fiber. TEDDY reported that intake of soluble fiber was not associated with the development of IA or T1D (60). However, Hakola et al. (61) found that in Finland increased early childhood gluten and dietary fiber intake were both associated with increased risk of IA, though they were not associated with T1D risk. A large nationwide cohort study in Norway confirmed the association between early childhood intake of gluten and a higher risk of T1D (62), but was not able to address the dietary fiber association.

Studies in normal volunteers show that introduction of gluten-free diets changes the composition of the microbiota (63). This gluten-induced dysbiosis may activate an inflammatory pathway that could lead to autoimmune diseases such as T1D (64, 65). There are no data in humans, but NOD mice on a gluten-free diet showed decreased *Bifidobacterium, Tanerella*, and *Barnesiella* species and increased *Akkermansia* species in feces, as well as a reduced diabetes incidence compared with mice not on a gluten-free diet (66).

### OMEGA-3 Fatty Acids

Two prospective cohorts of at-risk children (DAISY and DIPP) have shown that increased intake of omega-3 fatty acids (67, 68) or increased biomarker of omega-3 fatty acid status (67, 68) were linked to a reduced risk of IA. Omega-3 fatty acids are associated with alteration of the gut microbiota (69). A trial involving omega-3 fatty supplements for 8 weeks resulted in increases in the *Clostridiaceae, Sutterellaceae*, and *Akkermansiaceae* families, which then declined when supplementation was stopped (70). Trials in individuals at risk for metabolic syndrome (71) and with new onset type 2 diabetes (72) showed an impact of fatty acid supplementation on the composition of the microbiota. While it is not clear whether these changes would have an appreciable impact on IA or T1D risk, one can hypothesize that they may lead an increase in the production of anti-inflammatory compounds (69) that may affect the development of IA and T1D.

### Vitamin D and 25OHD

Vitamin D is obtained both from the diet and synthesis in the skin, and then metabolized to 25-hydroxyvitamin D (25OHD) in the liver. Higher plasma 25OHD levels were associated with lower risk of IA in both TEDDY (73) and in the TRIGR Ancillary Study (74), but not in other cohort studies of at-risk children (75–77). In children with IA, lower 25OHD levels were not associated with increased risk of progression to T1D (75, 78), suggesting that the protective role of vitamin D occurs early in the disease process. Vitamin D deficiency may contribute to autoimmunity via effects on the composition of the microbiota, although existing evidence is limited to animal studies or small human studies (79). Alternatively, vitamin D may act via direct effects on immune function rather than on the microbiota (80). Studies examining vitamin D intake and the gut microbiota have been inconsistent (81–83). A genome wide association study (GWAS) showed that the vitamin D receptor (*VDR*) gene was associated with beta diversity of the gut microbiota (84). How vitamin D affects the microbiota in this instance is not clear, although it is likely through an indirect mechanism as bacteria do not express the *VDR*.

### Total Sugar Intake and Dietary Glycemic Index

Increased intake of total sugars, and higher glycemic index of the diet are associated with increased risk of progression to T1D in autoantibody positive individuals (85, 86). A standard deviation (i.e., 49.7 g) increase in total sugar intake per day was associated with a 1.75-fold increased risk of progression to T1D after an average of 10.5 years (86). Sugar intake and glycemic index were not associated with initial development of IA, suggesting that these factors are only related to acceleration of the later stages of T1D. Potential mechanisms by which dietary carbohydrates and sugars may have an effect on T1D include metabolic (87, 88), and immunological pathways (89, 90), as well as pathways involving the microbiota, as reviewed in (91). Studies suggest that the high amount of simple sugars in our diet has lasting and detrimental effects on our microbiota (92–94). It is hypothesized that the replacement of microbiota-accessible carbohydrates (i.e., fiber) with the fats and simple carbohydrates that are characteristic of the Western diet may be negatively shaping the microbiota thereby leading to poor health outcomes.

## Uncovering Therapeutic Targets—A Proposed Roadmap Forward

### Approaches to Remodeling the Gut Microbiota for T1D Therapy

Several strategies are being considered to attempt to remodel the gut microbiota in T1D with an aim to improving immunological tolerance and beta cell function. Live bacteria (termed probiotics) can be introduced with a goal to either colonize the gut with a species that is absent, to increase the abundance of an existing species, or to provide beneficial metabolites without colonization. Alternatively, the goal may be to increase the overall community diversity by introducing a large bolus of diverse microorganisms with or without depletion of the existing microbiota as is the case with fecal microbiota transplantation (FMT). Another major approach under investigation is the use of “prebiotics” and diet-based therapy where the goal is to provide a food source to encourage the growth of beneficial bacteria. Alternatively, dietary supplements can be designed to directly deliver these bacterial metabolites themselves (95). The advantage of prebiotic approaches is that they do not rely on bacterial colonization, which may be ineffective if the T1D-associated intestinal environment is resistant to the introduction of beneficial bacteria. This has been shown to be the case in NOD mice, which were resistant to passive introduction of new species (96). T1D-associated genetic risk factors are thought to contribute to altering the intestinal immune environment, making it less hospitable to certain bacteria (38, 40, 97). Another approach is to identify the major modifiable lifestyle factors that lead to the establishment of a dysbiotic gut microbiota, but even this may need to be supported by one of the other strategies above, and lifestyle changes may be difficult to implement.

### Probiotics to Restore Microbial Dysbiosis in T1D

The notion of modulating gut homeostasis with supplementation of tolerogenic commensal microbes offers the potential for a safe method of intervention in the disease prevention setting. TEDDY reported an association between decreased risk of IA and early supplementation of probiotics (between the age of 0–27 days) in children with the highest risk HLA genotype (DR3/4), whereas the responses were highly variable in other age groups (98). These data suggest that early intervention with probiotics in infancy in T1D-susceptible populations, when the microbiota is being established, may be protective of future disease through probiotic colonization. However, this study did not look at dose or type of probiotic consumed, so more rational approach should be taken to identify disease protective probiotic strains. A number of *Lactobacillus* and *Bifidobacterium* species considered “Generally Regarded as Safe” (GRAS) microorganisms are commonly used in dietary supplements as probiotics worldwide and could potentially be trialed in T1D-susceptible newborns.

The use of probiotics for the prevention of T1D was initially tested with heat killed *Lactobacillus casei* BL21 in the NOD rodent model (99). It was found that the onset of diabetes was significantly higher in the control group than in the group that received *L. casei* (99). Similar results were obtained by weekly feeding the VSL#3 formulation to NOD rodents (100). VSL#3 is a formulation containing three species of *Bifidobacterium* (*B. longum, B. infantis*, and *B. breve*), four species of *Lactobacillus* (*L. acidophilus, L. paracasei, L. delbrueckii* subsp. bulgaricus, *and L. plantarum*), and *Streptococcus thermophilus*. The decrease in T1D onset was positively correlated with lower T cell infiltration in the pancreas in the VSL#3 treated group, suggesting a direct mechanistic role of the microbiota in T1D. It was also observed that VSL#3-treated mice was associated with increased IL-10 mRNA expression and with the presence of IL-10-positive infiltrating mononuclear cells (MNCs) in the pancreas. Clinical trials are underway to test the role of the VSL#3 probiotic formulation (Visbiome probiotic, ClinicalTrials.gov identifier NCT04141761, Table 1) on children and adolescents with T1D. In a randomized clinical trial designed to test for the prevention of allergy in a high-risk cohort, VSL#3 provided during the first 6 months of life was not related to the development of T1D (amongst *n* = 14 cases) or development of islet autoantibodies (amongst *n* = 25 cases) during the 13-year follow-up (101). However, as this study was not powered to investigate T1D these results should be viewed with caution. Furthermore, a drawback of this formulation is the inability to determine which of the strains within the formulation is the one exerting a beneficial effect.

**Table 1:** Clinical trials with completed or recruiting status with probiotic, prebiotics, and FMT interventions for T1D prevention or therapy.

**Clinical Trial identifier^a^**	**Status**	**Study population**	**Intervention**	**Sponsor**
**Probiotics**				
NCT03961854	Recruiting	T1D <1 Year; 8–17 years	Drug: *L. johnsonii* N6.2 Probiotic Drug: Placebo Capsule	University of Florida
NCT03961347	Recruiting	T1D <3 Years; 18–45 years	Drug: *L. johnsonii* N6.2 Probiotic Drug: Placebo Capsule	University of Florida
NCT03423589	Completed^b^	Full sibling of someone with T1D; 6–17 years	Dietary Supplement: VSL#3	Medical College of Wisconsin
NCT04141761	Recruiting	T1D <90 days; 6–17 years	Dietary Supplement: Visbiome (VSL#3) Other: Placebo	Medical College of Wisconsin
NCT03880760	Recruiting	T1D diagnosis; 6–18 years	Other: *L. johnsonii* MH-68, *B. animalis* subsp. lactis CP-9 and *L. salivarius* AP-32 Other: Placebo	China Medical University Hospital
**Prebiotics**				
NCT02442544	Active, not recruiting	T1D >1 year; 8–17 years	Dietary Supplement: Prebiotic (1:1 oligofructose: inulin) Dietary Supplement: Placebo	Alberta Children's Hospital
NCT04114357	Not yet recruiting	T1D >4 mos., <24 mos.; 12–16 years	Drug: Acetylated and Butyrylated High Amylose Maize Starch	Indiana University
NCT02903615	Active, not recruiting	T1D diagnosis; 18–70 years	Other: Novel diet (lower carbohydrate, Mediterranean-style, prebiotic fiber focus) Other: Standard diet (Standard diabetes diet recommendations)	Garvan Institute of Medical Research
ACTRN12618001391268	Completed^b^	T1D >6 months; 18–45 years	Dietary Supplement: Acetylated and Butyrylated High Amylose Maize Starch	Monash University
**Fecal transplant**				
NCT04124211	Recruiting	T1D diagnosis; 18–65 years	Biological: Fecal Microbiota Transplantation (FMT)	The Third Affiliated Hospital of Southern Medical University
NL3542	Completed (41)	T1D <6 weeks; 18–30 years	Biological: Fecal Microbiota Transplantation (FMT)	University of Amsterdam

* *Data from 08/09/2020*.

a *Identifiers starting with NCT were collected from clinicaltrials.gov; Identifier starting with ACTRN was collected from the Australia New Zealand Clinical Trials Registry and starting with NL from the Netherlands Trial Register*.

b *No results reported*.

Through interventions with different probiotics in different rodent models of disease, it has been observed that the mechanisms of action of probiotics are diverse and strain specific. The effects range from immunomodulatory to immunostimulatory (102). The strain specificity is usually overlooked; however, it is probably the biggest factor to consider in most animal and human studies looking to use probiotics for the prevention or treatment of a disease. In addition, many commercially available probiotic formulations are selected based on their robustness in industrial production and shelf life, instead of their beneficial effects on human subjects. Currently, many clinical trials are underway in order to be able to add health claims into their labels. It is possible that the variability observed on the TEDDY cohort may be due to the use of a diverse array of probiotic products not specifically selected for T1D prevention. Consequently, there is a significant need to identify bacterial strains that are associated with a beneficial health outcome. The availability of large microbiota datasets offers the possibility of (i) testing if GRAS microorganisms are negatively correlated with disease onset and (ii) isolate and test unconventional microbial species that may be used to prevent T1D onset.

#### L. Johnsonii N6.2 as a Proof-of-Concept Probiotic in T1D

As a proof of concept, Roche et al. performed a culture independent analysis of the bacteria in fecal samples collected from BB-DR and BB-DP rats (103). These experiments showed a significant difference in the intestinal microbiota of DR and DP rats. Valladares et al. (104) used this microbiota guided approach to isolate two strains of *Lactobacillus, L. johnsonii* N6.2 and *L. reuteri* TD1, that were abundant in BB-DR rodents, and subsequently administered the strains to BB-DP rats to test their role in T1D onset. It was found that *L. johnsonii* N6.2, but not *L. reuteri*, prevented diabetes onset when fed daily to BB-DP rats (104). Diabetes prevention correlated with a Th17 cell bias and elevated IL-23 levels within the mesenteric lymph nodes (105). Further *in vitro* studies indicated that the modification of dendritic cells (DCs) by oral feeding of *L. johnsonii* N6.2 contributed to the Th17 bias (105). It was also found that *L. johnsonii* N6.2 produces a metabolite that strongly inhibits the activity of the host enzyme Indoleamine 2,3-dioxygenase (IDO), *in vitro*. The substrate for IDO expressed by DCs is free tryptophan, a key regulator of T cell development and immune responses. Mass spectrometry analysis of the IDO catalytic heme-center supported the presence of a molecule in the cell-free culture supernatant of *L. johnsonii*, that modifies the prosthetic group of this immunoregulatory enzyme, and consequently modulates its activity. An *in vivo L. johnsonii* feeding assay performed in BB-DP rats showed *L. johnsonii* N6.2 induced lower levels of intestinal IDO gene transcription, which correlated with a decrease in the concentration of blood plasma kynurenine (106). Altogether, the authors suggest that this probiotic bacterium alters host IDO activity, with putative consequences on T cell development, intestinal physiology and ultimately T1D development.

Translating the use of *L. johnsonii* N6.2 toward the prevention of T1D in humans first required a pilot study in healthy individuals. Guidelines from the U.S. Food and Drug Administration (FDA) state that new probiotic strains tested in clinical trials with the intent to prevent or treat a disease are considered biologics (http://www.fda.gov/cber/guidelines.htm). As such, the safety, tolerability, and general response to consumption of this microorganism was tested in healthy individuals, where consumption of *L. johnsonii* N6.2 was found to be safe and well-tolerated (107). Results showed that *L. johnsonii* N6.2 can survive and may colonize the intestinal tract with minimal effects on the residing microbiota in healthy subjects. Significant changes in the kynurenine pathway metabolites, as well as innate and adaptive immune responses, were observed in serum and peripheral blood in the probiotic treatment group (107). Remarkably, the significant increase in tryptophan concentrations were positively correlated to a progressive increase in the cell counts of *Lactobacillus* over time in the probiotic group. Additionally, a significant increase in monocyte and NK cell frequencies correlated with a decrease in the percentage of CD4^+^ T cells. Changes were also observed in most CD8^+^ T cell subsets, including naïve (Tn), central memory (Tcm), effector memory (Tem) and effector memory expressing CD45RA (Temra). These results indicated that *L. johnsonii* N6.2 supplementation can evoke strain specific responses that may have the ability to modulate innate and adaptive immunity and potentially T1D progression that have not been previously reported for other probiotics (108–110). Altogether, the data collected thus far indicate that the mechanisms observed in animal models holds true in human subjects. Clinical trials are currently being performed to evaluate the safety of *L. johnsonii* N6.2 in children and adults with T1D as a stepping-stone for its translation into a preventive therapy for T1D (ClinicalTrials.gov identifiers NCT03961854 and NCT03961347, Table 1).

### Identification of Next-Generation Probiotics That May Reduce Progression of ISLET Autoimmunity

Most traditional probiotic bacteria originate from food, typically fermented dairy products or are previously isolated strains now currently used as commercial supplements. For example, Groele et al. plan to trial two common probiotic strains *Lactobacillus rhamnosus* GG and *Bifidobacterium lactis* Bb12 in new-onset T1D (111). Probiotic bacteria are not necessarily well-adapted to colonization and modulation of the human gut and other strains, termed “next-generation probiotics,” that are indigenous to the human microbiota may be more successful at inducing long-term benefits in T1D. When looking to identify which species should be introduced, it is important to consider the complex inter-relationships exist between species with different functional roles that support the growth of other community members. For example, one species may produce metabolites or substrates required to support the growth of another species, known as cross-feeding. One such interaction occurs when butyrate producers such as *Faecalibacterium prausnitzii* stimulate production of mucin by epithelial cells which in turn promotes the growth of mucinophiles such as *Akkermansia*. Mucin degradation and fermentation by *Akkermansia* releases oligosaccharides and acetate which in turn further supports the growth of butyrate producers in a feedback loop (112). This has led to the concept that increasing the abundance of a single species such as *Akkermansia* could then enhance the abundance of other beneficial species resulting in a broader beneficial impact. Indeed, some studies have suggested that a reduction in *Akkermansia* may be associated with IA or T1D (22, 58). This concept was tested by introduction of *Akkermansia muciniphila* by oral gavage into NOD mice resulting in an increase in mucin production by goblet cells in the colon, while reduced expression of the inflammatory marker Emr1 and increased expression of the anti-inflammatory marker Ym1 was also observed (113). The combined effect of these changes included increasing immune regulatory factors (increased IL-10 and TGF-beta in the pancreatic LN and Tregs in the islets) and delayed diabetes onset. It is likely that many more species with similar functions may also protect from T1D, at least in animal models. Recent progress in the ability to monoculture many more members of the gut microbiota community means that efforts are now ongoing aiming to systematically screen for taxa that can prevent T1D. These efforts may then identify candidates which can be tested for beneficial effect in human T1D.

### Fecal Microbiota Transplantation (FMT)

FMT is the practice of transferring the total bacterial population from a healthy donor stool sample (either by rectal or oral routes into the gastrointestinal tract) with a goal of remodeling the recipient's microbiota for therapeutic benefit. This approach has gained traction due to dramatic benefits found using FMT for treatment of recurrent *Clostridium difficile* infection (114). In *C. difficile*, FMT was shown to significantly increase the diversity of the recipient's microbiota 2-weeks after transfer. Using genetic approaches to track donor derived microbial strains, Li et al. showed that donor derived bacteria persisted for 3-months after FMT (115). However, colonization was more successful for species that were already present in the recipient's own microbiota, supporting that host compatibility is a barrier to introduction of new taxa. In a study of individuals with metabolic syndrome, FMT from lean donors induced short-term changes in the recipients gut microbiota composition and improved insulin sensitivity and decreased glycated hemoglobin levels at 6 weeks (116). Taxa that were increased at 6 weeks included acetate producer *Bifidobacterium pseudolongum*, lactate-producing *Lactobacillus salivarius*, butyrate-producing *Butyrivibrio, Clostridium symbiosum*, and *Eubacterium* species. This suggests that modest benefits to glycemic control can be gained by FMT induced remodeling of the gut microbiota, but that strategies to increase the durability of the effects are needed.

The first study of FMT in T1D was recently published (41). Nieuwdorp and colleagues completed a trial of allogeneic vs. autologous FMT in individuals with new-onset T1D (Netherlands Trial Register identifier NL3542, Table 1). They found that the participants that received back their own autologous stool sample had preserved beta cell function (stimulated C-peptide) compared with those that received FMT from an allogeneic healthy donor (41), and this was correlated with certain microbial metabolites. This may indicate that the microbiota from the subject's own sample were better adapted to re-colonize than species from a non-diabetic donor. Results from this study demonstrate that FMT and microbiota-targeted therapy in general holds promise for promoting immune tolerance and preserving beta cell function in T1D. In the long-term, it is unlikely that FMT in its current form will progress to a wide-spread therapy for T1D due to both safety and palatability considerations. Recently, two cases of drug-resistant *E. coli* bacteraemia occurred following FMT in two separate clinical trials resulting in the death of one immunocompromised patient (117). It is more likely that if FMT demonstrates therapeutic benefit, knowledge from these studies will lead to the development of defined microbial consortium-transfer based approaches. The challenge will be in how to tailor such products to account for compatibility with core microbiota present in each recipient that together with diet and genetic background strongly influence donor engraftment (115).

### Prebiotics and Diet-Based Approaches– Providing the Nutritional Landscape for Beneficial Bacteria

The use of diet-based therapy to target the gut microbiota can circumvent the need for engraftment of donor microbes into the system. These include prebiotics, which are defined as “a substrate that is selectively utilized by host microorganisms conferring a health benefit” (118). These include certain oligosaccharides (including human milk oligosaccharides) and fermentable fibers. Using such an approach, Ho et al. delivered a prebiotic, oligofructose-enriched inulin or placebo, for 12 weeks in children that had T1D for at least 1 year (119). At the completion of the diet (3 months), the prebiotic group (*n* = 17) had significant preservation of c-peptide compared to the placebo group (*n* = 21), though this benefit was not sustained at 6 months after the diet was stopped. The prebiotic administration was associated with a significant increase in the *Bifidobacterium* genera. Other approaches introduce specific modifications to prebiotics to increase the delivery of beneficial metabolites such as SCFAs. For example, high amylose maize starch (HAMS) is a resistant starch that can be modified by acetylation (HAMSA) and/or butyrylation (HAMSB). When HAMSA or HAMSB are fermented in the colon, large quantities of acetate and butyrate are released. In NOD mice, a HAMSAB diet provided before disease onset almost completely prevented progression to autoimmune diabetes (95). Protection was associated with expansion of regulatory T cells and a decreased frequency of islet-specific effector T cells via a mechanism dependent on remodeling of the gut microbiota. HAMSAB is being tested in adults with established T1D in the “TOGeTHER” (*T*ype One *G*ut *Ther*apy) trial (Australia New Zealand Clinical Trials Registry identifier ACTRN12618001391268, Table 1) for its potential to increase systemic acetate and butyrate delivery and modulate the microbiota, immune tolerance and glycemic control. Similarly, HAMSAB is being tested in new-onset T1D in a randomized cross-over trial (NCT04114357). In a related approach, de Groot et al. gave oral sodium butyrate to adults with established T1D for 1 month (120). Unlike HAMSAB, which is utilized in the colon by the gut microbiota releasing acetate and butyrate, oral sodium butyrate capsules deliver butyrate mainly to the small intestine. In this study, oral butyrate delivery did not lead to increased butyrate availability in the colon, nor did it impact peripheral innate immunity, islet-specific T cell autoimmunity or beta cell function. However, this mode of butyrate delivery, dose, or duration of treatment may not have been appropriate to achieve the disease protective effects seen in animal studies. In studies in healthy subjects, three different resistant starches: maize, potato, and tapioca-based starches with different chemical cross-linking, were compared in a dose-escalation study with a non-resistant (digestible) control starch (121). The authors found that the crystalline maize resistant starch increased butyrate production together with *Eubacterium rectale* (a major butyrate producer), while the cross-linked tapioca resistant starch increased the SCFA propionate and *Parabacteroides distasonis*. *Parabacteroides distasonis* produces succinate, which is then converted to propionate by other bacteria. Neither the potato-based resistant starch nor the control starch increased SCFA concentrations. These data suggest that specialized dietary fibers can be used to target specific SCFA, and those that favor butyrate production could be specifically evaluated in T1D.

### New Directions in Microbiota Targeted Therapy for T1D

While much work is focusing on ways to increase SCFA production by the gut bacteria in T1D, many other microbial pathways and bioactive compounds produced by commensal bacteria may be beneficial for immune tolerance and glucose homeostasis. For example, the secondary bile acids isoDCA, isoalloLCA, and lithocholic/3-oxo-lithocholic acids, which are metabolized by bacteria in the colon from primary bile acid waste, have been shown to promote the generation of peripheral Tregs in the colon (122–124). Supplementation of these secondary bile acids was achieved in mice by either delivering them in drinking water, incorporating them into the mouse chow or by transferring a consortium of bacteria engineered with the metabolic capability to produce isoDCA. Thus, translation of such approaches to humans may have potential to reduce intestinal inflammation and potentially autoimmunity. Another example are microbe derived metabolites derived from breakdown of the amino acid tryptophan into indoles. These indoles are ligands for the aryl hydrocarbon receptor (AhR) which then promotes gut barrier integrity, Tregs and IL-22 production. In mouse models of T1D, oral administration of AhR ligands have been shown to expand Tregs, reduce insulitis and diabetes progression (125, 126). The beneficial gut bacteria *Faecalibacterium prausnitzii* is a high butyrate producer, but also produces an anti-inflammatory protein called MAM which inhibits NFκB activation and alleviates chemically-induced colitis (127). Identification of anti-inflammatory molecules produced by gut bacteria is currently a rich field, and it is likely that many candidates will be identified with promise for human disease modulation including T1D. Such molecules (now termed “postbiotics”) could be delivered as stand-alone drugs or the bacteria that produce these molecules could be targeted for expansion by prebiotics or probiotic based therapies.

While microbiota-based therapies hold much potential, it is quite likely that combination therapy approaches will be needed, especially at disease onset when autoimmunity and beta cell destruction is well-advanced. In cancer, it has recently been shown that the efficacy of check-point inhibitor immunotherapy relies on the gut microbiota (128, 129). At least in mouse models, co-delivery of a probiotic cocktail of *Bifidobacterium* strains improved the outcome of anti-PDL1 cancer therapy (130) and likewise *Bacteroides thetaiotaomicron* or *Bacteroides fragilis* transfer enhanced anti-CTLA-4 cancer therapy (128). There is good rationale that such combination approaches may also be of benefit in T1D. For example, low-dose IL-2 therapy, which protects NOD mice from diabetes (131) and is currently being trialed in human T1D, expanded Treg in the gut mucosa and led to an altered gut microbiota in NOD mice (40). Therefore, there may well be synergy between microbiota targeted and IL-2 based therapy. Similarly, in a rat model of T1D, two immunotherapies which prevent disease: the histone deacetylase inhibitor ITF-2357 and IL-1 receptor antagonist (Anakinra) also modify the gut microbiota. Using this rationale, Chan et al. showed that combining anti-CD3 immunotherapy with a prebiotic oligofructose increased the rate of disease reversal when treatment was started at the onset of hyperglycemia (132). In a more sophisticated approach, Takiishi et al. have used a probiotic strain of *Lactococcus lactis* modified to secrete the autoantigen proinsulin and IL-10 in combination with anti-CD3 therapy (133, 134). They showed that this combination therapy was more effective than either treatment alone in reversing disease at the onset of hyperglycemia in NOD mice. Hence, combination therapies targeting multiple disease pathways, including via the gut microbiota, could prove beneficial for treatment of T1D.

## Highlights and Future Directions

Taken together, these studies provide evidence that the diet and other modifiers of the microbiota can alter T1D autoimmunity. While there remain a number of gaps in determining the exact role for these factors, the evidence suggests an important function. Furthermore, while insulin has been around for 100 years, there are no other treatments for T1D and other approaches are urgently needed. Modifying the microbiota through diet, probiotics, prebiotics or some combination thereof, is an attractive opportunity for disease modification, especially in very young children and/or in primary prevention, where immunotherapies and other therapeutic approaches may have limited accessibility.

Several of the dietary factors that have been associated with either IA or T1D appear to have some influence on the gut microbiota. In order to determine if the mechanism by which diet impacts T1D is through changing the microbiota, one would need to conduct a true causal study. However, thus far, the majority of data to date are all indirect, and the limited clinical trials in this area have yet to show causality. Needless to say, there is sufficient evidence to suggest that clinical studies applying these approaches are both feasible and warranted, given the correlative evidence, the relative safety of such approaches and the need for disease modification in T1D.

**Figure 1:**
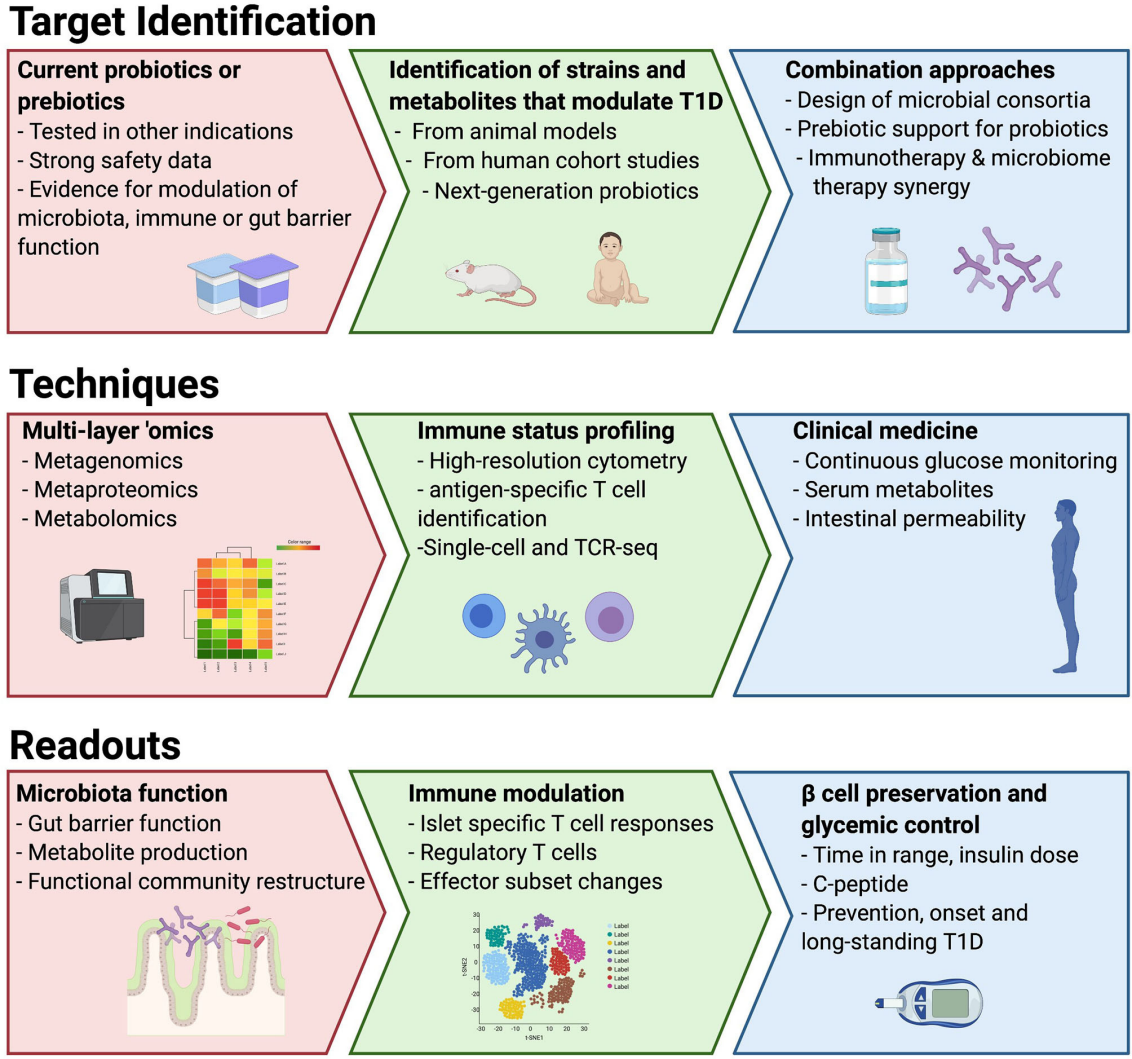
Proposed roadmap to utilize different approaches, model systems and techniques to drive to microbiota-targeted therapeutics in T1D.

To date, most of the studies of the microbiota in T1D have used stool bacterial composition as a surrogate to understand functional changes in the microbiota, the intestinal environment and immune system. Moreover, few studies have attempted to understand the direct role the microbiota may be playing in destruction of the beta cells, such as those mechanisms that might be in play due to the presence of a leaky gut in T1D linked to microbial dysbiosis (27, 28). To overcome these limitations, the use of well-designed preclinical models as well as quick entry into clinical studies for interventions with good safety profiles should be prioritized (Figure 1). In this way, dietary studies may be the most straightforward interventions, but they are fraught with limitations with respect to adherence. We suggest two parallel approaches to probiotic interventions in T1D: (1) identification and isolation of strains and/or their metabolites important in IA or T1D and (2) testing commercial probiotics with claimed benefits in other immune-based diseases. The use of integrated “omic” technologies can greatly advance our ability to functionally assess the impact of new therapies on the complex gut ecosystem. The benefits of metagenomics have been widely realized to uncover bacterial pathways encoded in the microbiota that may be altered in disease (21). Metaproteomics is an emerging technology with potential to measure the actual functional activity of both the microbes and the host by measuring stool proteins derived from bacteria, the gut and the pancreas (29). Metabolomics of serum has been used to reveal differences in lipid metabolism and nutrient uptake in children prior to the development of autoimmunity (135, 136). As some plasma metabolites can be derived from the gut microbiota, a direct measurement of microbiota metabolite production in stool would help to understand whether circulating metabolites altered in T1D are microbiota derived. Fecal metabolomics has been successfully used to elucidate distinct disease-related microbiota signatures in other diseases (137, 138), but similar studies in T1D are not yet available. Finally, as the evidence suggests in other diseases, microbiota-based approaches may have an added benefit in traditional therapeutics and should be considered as adjunctive therapy.

## Author Contributions

JLD, EEH-W, GLL, and JMN conceived the concept and co-wrote the manuscript. All authors contributed to the article and approved the submitted version.

## Conflict of Interest

JLD is an employee of Janssen Research and Development. The remaining authors declare that the research was conducted in the absence of any commercial or financial relationships that could be construed as a potential conflict of interest.
